# CoESPRIT: A Library-Based Construct Screening Method for Identification and Expression of Soluble Protein Complexes

**DOI:** 10.1371/journal.pone.0016261

**Published:** 2011-02-22

**Authors:** Yingfeng An, Patrick Meresse, Philippe J. Mas, Darren J. Hart

**Affiliations:** 1 Grenoble Outstation, European Molecular Biology Laboratory, BP181, Grenoble, France; 2 Unit of Virus Host-Cell Interactions, UJF-EMBL-CNRS, UMI3265, Grenoble, France; University of Queensland, Australia

## Abstract

Structural and biophysical studies of protein complexes require multi-milligram quantities of soluble material. Subunits are often unstable when expressed separately so co-expression strategies are commonly employed since *in vivo* complex formation can provide stabilising effects. Defining constructs for subunit co-expression experiments is difficult if the proteins are poorly understood. Even more problematic is when subunit polypeptide chains co-fold since individually they do not have predictable domains. We have developed CoESPRIT, a modified version of the ESPRIT random library construct screen used previously on single proteins, to express soluble protein complexes. A random library of target constructs is screened against a fixed bait protein to identify stable complexes. In a proof-of-principle study, C-terminal fragments of the influenza polymerase PB2 subunit containing folded domains were isolated using importin alpha as bait. Separately, a C-terminal fragment of the PB1 subunit was used as bait to trap N-terminal fragments of PB2 resulting in co-folded complexes. Subsequent expression of the target protein without the bait indicates whether the target is independently stable, or co-folds with its partner. This highly automated method provides an efficient strategy for obtaining recombinant protein complexes at yields compatible with structural, biophysical and functional studies.

## Introduction

Many proteins do not function as monomers in the cell, but interact with partners in stable or transient complexes. Therefore, to understand their function, characterisation of subcomplexes of multi-component entities is necessary [1]–[4]. Characterisation of protein complexes has received considerable attention in the post-genomic era and large scale experimental and bioinformatic studies have identified the subunit content of many protein complexes. These subunits exist in a continuum from completely unstructured proteins that fold upon binding to those that fold individually and subsequently dock together [5]–[10]. Although the components of many protein complexes have been catalogued using proteomics methods (e.g. [11]), recombinant expression of intact complexes for structural studies remains a major challenge. In particular, careful experimental validation of complexes predicted from high throughput studies is necessary to filter out transient, unstable or non-existent complexes prior to commencement of recombinant expression trials [12].

A common strategy for obtaining protein complexes is to express single proteins separately and then reconstitute complexes from purified components. Various experimental approaches for assembling protein complexes under *in vitro* conditions have been developed [13]–[15]. Although these methods can be efficient, the formation of protein complexes is dependent on soluble expression of each component. In many cases when heterologous expression systems are employed, complex subunits cannot fold in the absence of their partners and so co-expression strategies are employed to produce subunits in the same host cell [16], [17]. Co-expression facilitates soluble complex formation by allowing co-folding or stabilisation through binding of protein partners. This can reduce or prevent aggregation or degradation, and alleviates the need for *in vitro* purification and reconstitution [18]. Several studies have revealed how co-expression can perform better than reconstitution from separately purified components [4], [19].

Among various systems to produce heterologous proteins for structural and functional studies, protein expression in *Escherichia coli* is the most commonly used system because it is genetically simple, inexpensive for producing large quantities of proteins and permits the isotopic or heavy atom labelling of proteins that is necessary for some structural methods. However, when full-length eukaryotic proteins are produced in *E. coli*, aggregation and insolubility problems often arise resulting in low yields [20]. Contributing factors include large size, susceptibility to proteases, intrinsic segmental flexibility or requirements for post-translational modifications. In fields such as structural biology, expression of more stable sub-full-length protein constructs is a common strategy, but this necessitates prediction of domain boundaries in order to design constructs. Multiple sequence alignments are the most common tool for domain prediction and are used to guide subsequent trial-and-error PCR subcloning experiments. One problem with this approach is that many proteins are poorly understood and have no significant sequence similarity with others, precluding this approach. In these cases secondary structure predictions and order/disorder predictors can help identify folded domains. Several convenient meta server tools exist that combine different secondary structure and order predictors with additional information sources to provide more accurate domain predictions and even associated automated primer design, for example ProteinCCD [21] and the SGC Domain Boundary Analyser [22]. Such tools can be very valuable, but do not always result in successful expression, in part because they are generally low resolution and even small variations at the edges of construct can affect the level of expression and stability of the products in an unpredictable manner.

For such problematic targets, a number of random library-based strategies have been developed that generate large collections of randomly truncated or fragmented constructs and couple these to a screen or selection process to identify rare soluble clones [23]–[28] reviewed in [29]–[31]. The ESPRIT technology developed in our laboratory uses exonuclease III/mung bean nuclease protocols [32] to generate unidirectionally or bidirectionally truncated construct libraries. Tens of thousands of clones can then be screened in a colony array format using efficiency of *in vivo* biotinylation of a fused biotin acceptor peptide to enrich soluble clones from the library [28]. Positive clones are then further validated in 96 well plates by automated affinity chromatography purification [33]. ESPRIT has been used to express a number of challenging proteins for further structural study [27], [34]–[39].

There has been no detailed description of library methods being used to express protein complexes directly i.e. incorporating co-expression approaches. Here, we have established a high-throughput automated strategy in which a library of constructs is screened for solubility in the presence of an interacting bait protein. As such, it is similar in concept to two-hybrid methods but in the context of recombinant over-expression of multi-milligram quantities of material required for many downstream applications including structural biology and vaccine research. Soluble protein complexes identified by this method can either result from association of pre-folded partners or inter-folded polypeptide chains. To demonstrate the isolation of both types of complexes, we used subunits from the heterotrimeric influenza RNA polymerase that comprises three subunits: PA, PB1 and PB2. This complex catalyses the transcription and replication of the viral RNA genome in the nucleus of infected cells [40]. The PB2 subunit has been shown to interact with importin α to achieve nuclear localisation [27]. For many years the polymerase subunits resisted all attempts at soluble recombinant expression due their relatively large sizes (PA: 716 aa; PB1: 757aa; PB2: 759aa) and their lack of homology with other proteins which prevented domain identification using multiple sequence alignments. The PB2 subunit was previously studied using the ESPRIT method leading to the expression and subsequent structure solution of a series of novel domains key to viral function [27], [37], [39] reviewed in [41], [42].

Here we screened PB2 gene libraries against bait proteins with the aim of isolating purifiable complexes. Firstly a 5′ truncation library of the gene encoding the polymerase PB2 subunit was screened against importin α1 that has been shown to bind the purified C terminus of PB2 when mixed *in vitro* [39]. Secondly a 3′ truncation library of the same subunit was screened against a poorly behaving, marginally soluble C-terminal construct isolated from the PB1 polymerase subunit in an earlier ESPRIT experiment (data not shown). A similar PB1 construct was recently shown by X-ray crystallography to form an inter-folded complex with a short N-terminal fragment of PB2 [43], explaining its poor behaviour in isolation. In both experiments, a series of soluble complexes were isolated, some of which were similar to structurally validated forms, while others may be of potential interest in future functional studies.

The application of ESPRIT in this co-expression format (CoESPRIT) provides a powerful way of identifying well-expressing soluble complexes for *in vitro* and *in vivo* biochemical and structural characterisation, as well as immunisation, high throughput screening and other applications that require multi-milligram quantities of material. Additionally the same format has the potential for co-expression of other interacting proteins such as chaperones and modifying enzymes, widening the repertoire of expression tools for obtaining sufficient quantities of purified protein complexes.

## Results

### Design of a plasmid system allowing library construction and bait co-expression

The plasmids used for library construction are pET9a derived, encoding the PB2 gene fused to either 5′ hexahistidine tag or 3′ biotin acceptor peptide coding sequences with, in each case, the other tag present out-of-frame at the opposing end and separated from the coding sequence by a pair of restriction sites enabling unidirectional truncation [32]. The plasmid pYAN008 for N-terminal truncation libraries encodes an in-frame fusion of biotin acceptor peptide and an out-of-frame fusion of the hexahistidine tag sequence with the PB2 gene; while the plasmid pYAN009 for C-terminal truncation libraries encodes an out-of-frame fusion of biotin acceptor peptide and an in-frame fusion of the hexahistidine tag sequence with the PB2 gene (Fig. S1A). Following the exonuclease III deletion procedure and religation, one third of plasmid constructs encode the PB2 insert in-frame with both N- and C-terminal tags.

The co-expression vector for the fixed bait protein contains several key features (Fig. S1B). Firstly the RIL plasmid provides replicative compatibility with the pET library vector and supplements rare tRNAs. Secondly, a T7 expression cassette was introduced previously (see methods) resulting in a vector, pLIC-SGC1, for co-expression purposes. The advantage of using two separate plasmids is that one library can be screened against multiple baits in parallel, as performed here when the PB2 C-terminal truncation library was analysed for interaction with three different C-terminal constructs of its partner PB1. Thirdly, a FLAG tag fused to the N terminus of the bait (importin α1 or PB1 C-terminal sub-constructs) permits its identification after the IMAC purification of the library protein.

**Figure 1:**
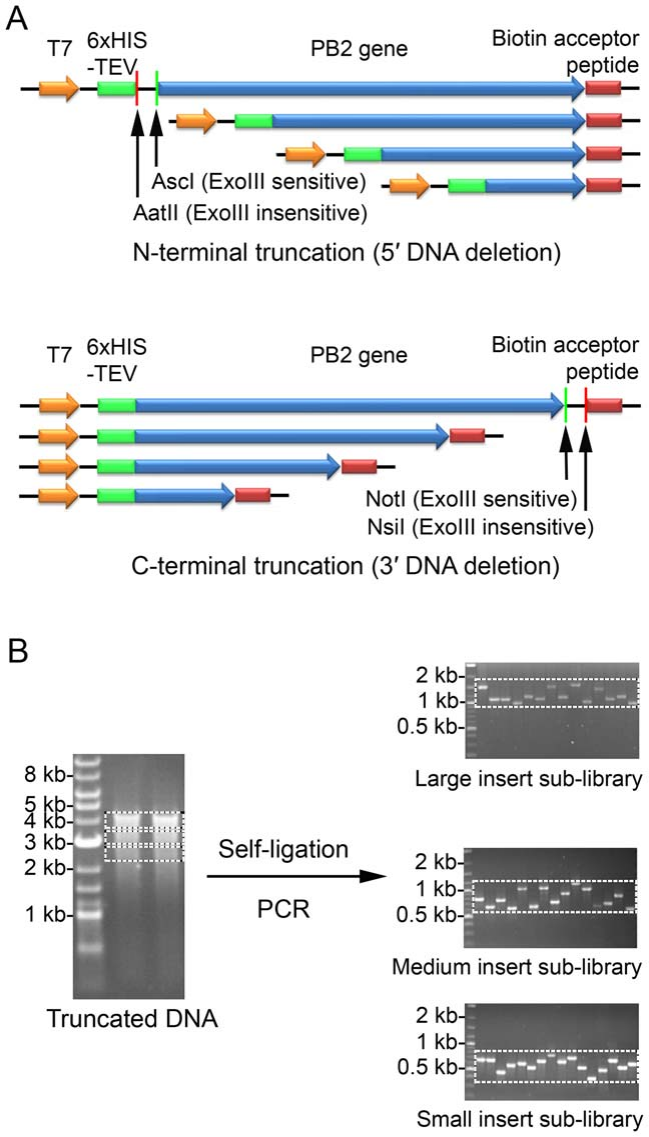
Synthesis of incremental truncation libraries of the PB2 gene for complex screening. (A) Plasmid DNA containing a precloned PB2 gene insert is linearised with a pair of restriction enzymes: one that produces an exonuclease III resistant 3′ overhang (*Aat*II or *Nsi*I), the other a 5′ overhang which is as substrate for exonuclease III (*Asc*I or *Not*I). A time course digestion is performed to produce a set of evenly truncated constructs. Mung bean nuclease and *Pfu* polymerase are then used to generate blunt ligatable ends; (B) DNA fragments are separated by agarose gel electrophoresis and excised in three size ranges. After ligation, the sublibraries are recovered by transformation of *E. coli* and insert size assessed by colony PCR with flanking primers. Pooled plasmid DNA is then used to transform an expression strain that co-expresses the bait protein.

Comprehensive N-terminal and C-terminal truncation libraries were generated using an exonuclease III and mung bean nuclease protocol (Fig. 1A) by cutting the pair of restriction sites at the end of the insert to be truncated. The library was divided into sub-libraries by size selection on agarose gel that were treated independently, thereby separating potentially dominant small fragments from less well expressing, but perhaps more interesting larger constructs in the subsequent screening step. Colony PCR and DNA sequencing of inserts from recovered clones after ligation and transformation demonstrated an even distribution of construct sizes (Fig. 1B). These sub-libraries were then used to transform *E. coli* BL21 (DE3) that had been prepared as competent cells containing the bait plasmid.

### Two step screen for expression of soluble complexes

**Figure 2:**
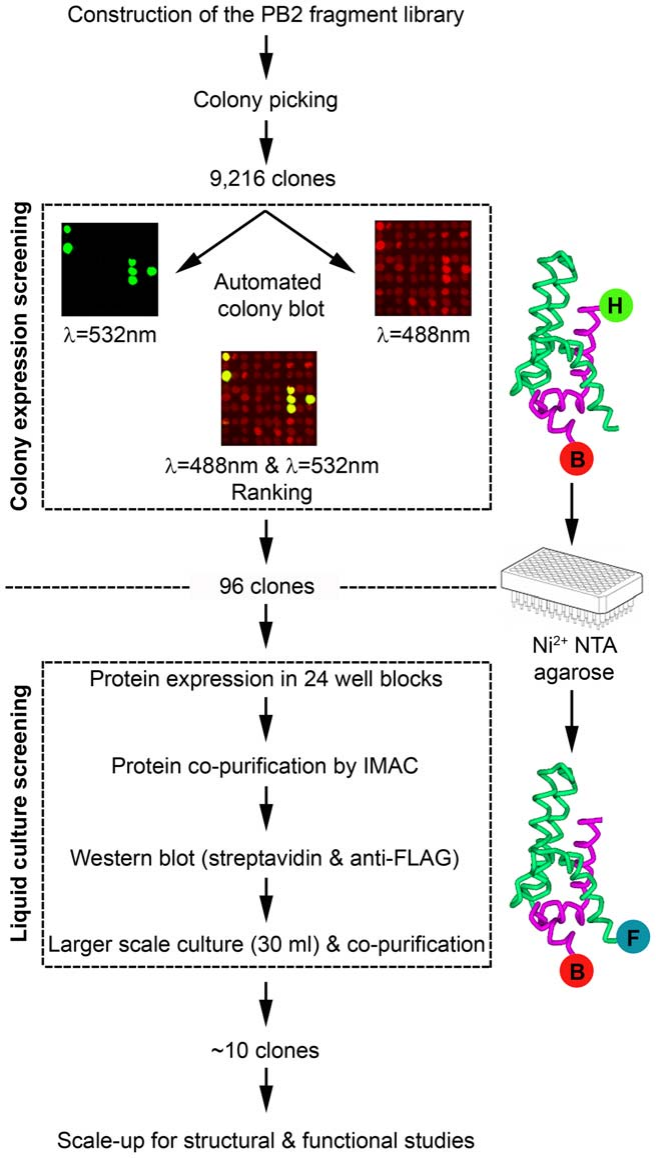
Process of screening for soluble protein complexes from truncation libraries. 9,216 colonies were picked for each library and gridded onto nitrocellulose membranes over agar to generate colony arrays in which protein expression was induced. Expression levels and putative solubility were assessed by intensity of PB2 N-terminal hexahistidine tag (green) and C-terminal biotinylated biotin acceptor peptide (red) signals respectively on the colony blot. The 96 highest ranked clones were expressed in 4 ml liquid expression cultures, purified by Ni^2+^NTA affinity chromatography and eluted fractions analysed by western blot to identify bait FLAG tag (blue) and PB2 biotinylation signals (red). From these data, a panel of clones was selected for 30 ml scale expression tests, then further prioritised for larger scale production.

The libraries were screened using standard colony picking and liquid handling robots in a work flow designed to identify any forms of the soluble library targets and, where found, determine whether they bound the bait protein (Fig. 2). In a first step of the screen, the library was clonally separated by colony picking and gridded on to nitrocellulose membranes according to the ESPRIT method for single proteins [27], [28]. In total, 9,216 transformant colonies from each library were isolated representing an approximate four-fold oversampling of constructs calculated from the length of the gene (2,277 bp) and the frequency of truncated inserts. The exonuclease protocol for generating inserts is therefore able to generate many times more constructs than would be possible using a classic PCR cloning strategy, but requires the extra step of colony picking to isolate individual clones. Using a solid pin arrayer, the sub-libraries were printed as inocula onto nitrocellulose membranes over agar to generate colony arrays where protein expression was induced by transfer of membranes to IPTG-containing inducing agar. The colonies were lysed *in situ* using sodium hydroxide soaked filter paper that denatures all the cellular proteins and adsorbs them to the membrane [44]. These were then hybridised with fluorescent conjugates of streptavidin against the C-terminal biotin acceptor peptide, and a secondary antibody recognising the anti-hexahistidine monoclonal antibody against the N-terminal affinity tag.

Values of hexahistidine and biotinylation signal intensities were extracted from arrays and their distribution determined. The weak background biotinylation signal observed for all colonies is a consequence of the endogenous biotinylated protein BCCP [45] and is useful for guiding the array fitting software. To obtain a list of putative solubly expressing clones for further investigation in the second liquid expression and purification step of the procedure, only colonies exhibiting visible hexahistidine tag signals were selected, thus eliminating truncated or degraded protein products that, even if soluble, would not be purifiable (Fig. 2). Based upon this, the most intense 96 positive clones with significantly higher biotinylation signals than background were extracted from the large bank of clones (Fig. S2). Ranking of clones for their *in vivo* biotinylation efficiency permits enrichment of a population of soluble expression clones from the library since soluble constructs (where they exist) are generally better substrates for this cytoplasmic post translation modification than degraded or insoluble targets, and therefore appear higher in the ranking list. However, the desired solubility phenotype itself is imperfect: in some cases the identified clones may express completely solubly [27], but we have often observed that some of the best constructs found for challenging targets show a mixed phenotype with expressed material in both insoluble and soluble fractions with the latter the minor component [38]. Scale up of partially soluble ESPRIT hits to larger culture volumes [38] or lysis buffer and strain optimisation [46] can overcome these problems to yield sufficient quantities for downstream studies.

A summary of the processing of each library showing the number of clones passing through each step is presented in Fig. S3. Ninety-six of the most intensely biotinylated, hexahistidine-positive clones from each of the four libraries were grown in 4 ml volumes and protein expression induced. After robot assisted purification in 96 well filter plates charged with Ni^2+^NTA agarose [19], purified fractions were obtained by elution in imidazole buffer. Since only the PB2 constructs carry an N-terminal hexahistidine tag, retention of the bait protein on the Ni^2+^NTA affinity column depends on its interaction with this target. Complex formation was determined using a combined streptavidin and anti-FLAG antibody western blot to confirm the presence of the soluble target protein and co-purifying bait respectively.

**Figure 3:**
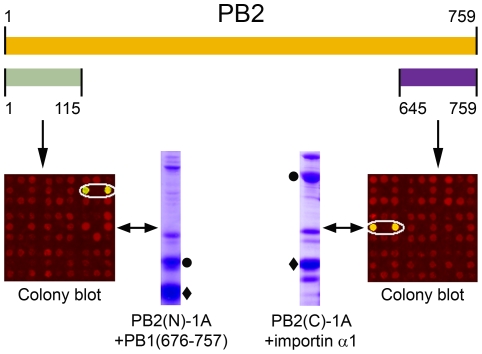
Colony signals and subsequent 4 ml purification test of highest ranking biotinylated clones. After colony screening, the clones exhibiting the strongest signals of biotinylation for each library, PB2(N)-1A and PB2(C)-1A, were also the best ones during the subsequent liquid culture testing. PB2 fragments are marked with a black diamond and the bait protein, PB1 (amino acids 676–757) or importin α1, with a black circle.

For both the PB2 C-terminal truncation library screened against PB1-(676–757) and the PB2 N-terminal truncation library screened against importin α1, most constructs generated purified PB2-bait complexes strongly visible by streptavidin-western blot of the purified fractions (74 and 95% respectively; Fig. S3). This was due both to an abundance of soluble solutions and also the 3-fold oversampling resulting in many PB2 constructs of similar sizes. For both libraries, only a subset of the hits were processed further through medium scale purification and constructs representing different sizes were prioritised over similar sized hits. Of this set of clones, those with strongest biotinylation signals during colony screening were also the best ones during the subsequent liquid culture testing (Fig. 3). Thus, although protein expression in colony and liquid states might be expected to differ, the approach of ranking the former to predict the latter therefore seems effective and provides a useful indicator of the amount of soluble protein likely to be produced downstream.

By contrast, when the PB2 C-terminal truncation library was screened against alternative PB1 fragments (576–757; 586–696) only faint PB2 bands were observed by streptavidin-western blot after Ni^2+^ NTA purifications under identical fluorescent scanner parameters, with no evidence of complexes. Eight and four clones respectively were selected for medium scale purification studies (Fig. S3) but yielded no purified material; these libraries were not studied further.

### Medium scale purification of complexes

**Figure 4:**
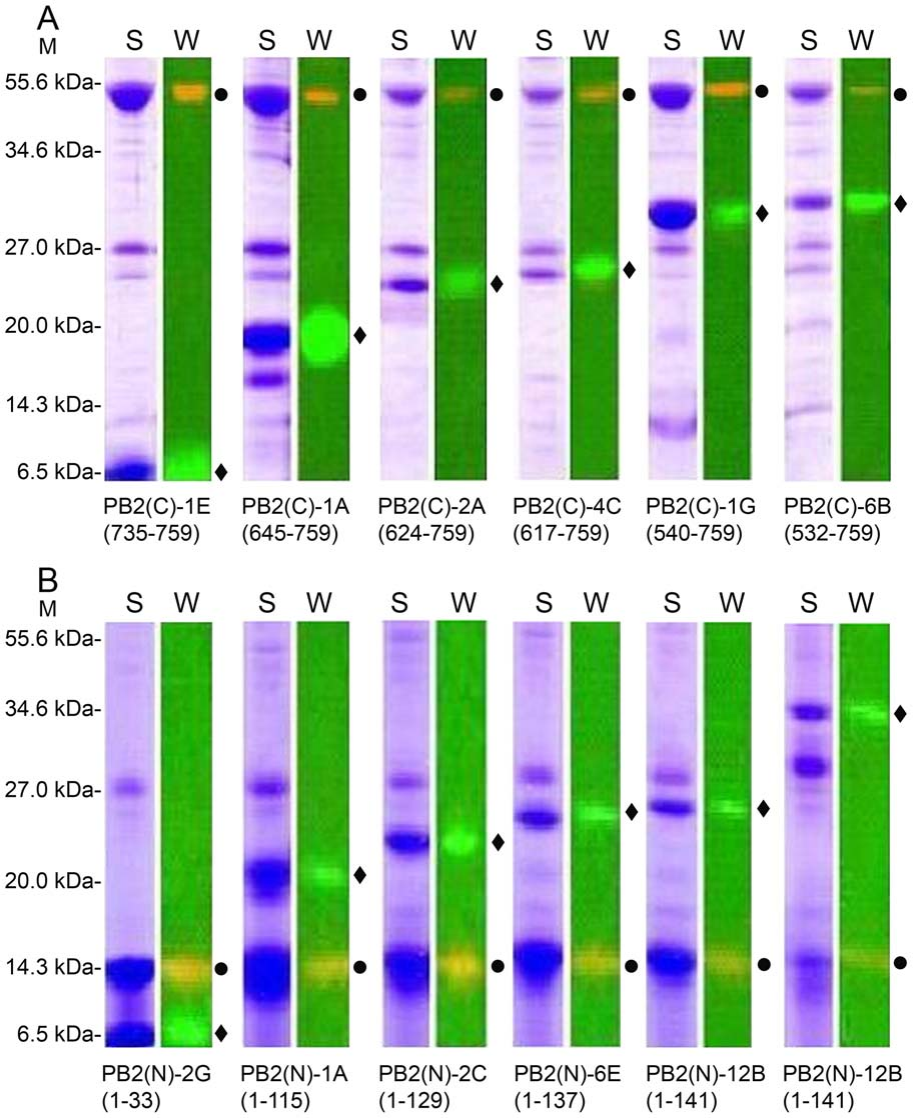
Co-expression and co-purification of PB2 protein complexes. Purified complexes from 4 ml expression trials with western blot confirmation of protein identities for (A) importin α1 bait screened against PB2 N-terminal truncation libraries and (B) PB1 (amino acids 676–757) bait against PB2 C-terminal truncation libraries. Results are presented for the six best clones from each library. Annotations are: M, molecular weight marker; S, SDS-PAGE gel; W, western blot. Purified proteins were stained with Coomassie blue after SDS-PAGE. For western blots, bait proteins were detected by anti-FLAG monoclonal antibody with bands in red (black circle). PB2 library proteins were detected by streptavidin binding and are green (black diamond). Numbers in parentheses are the amino acids of the PB2 library protein.

**Figure 5:**
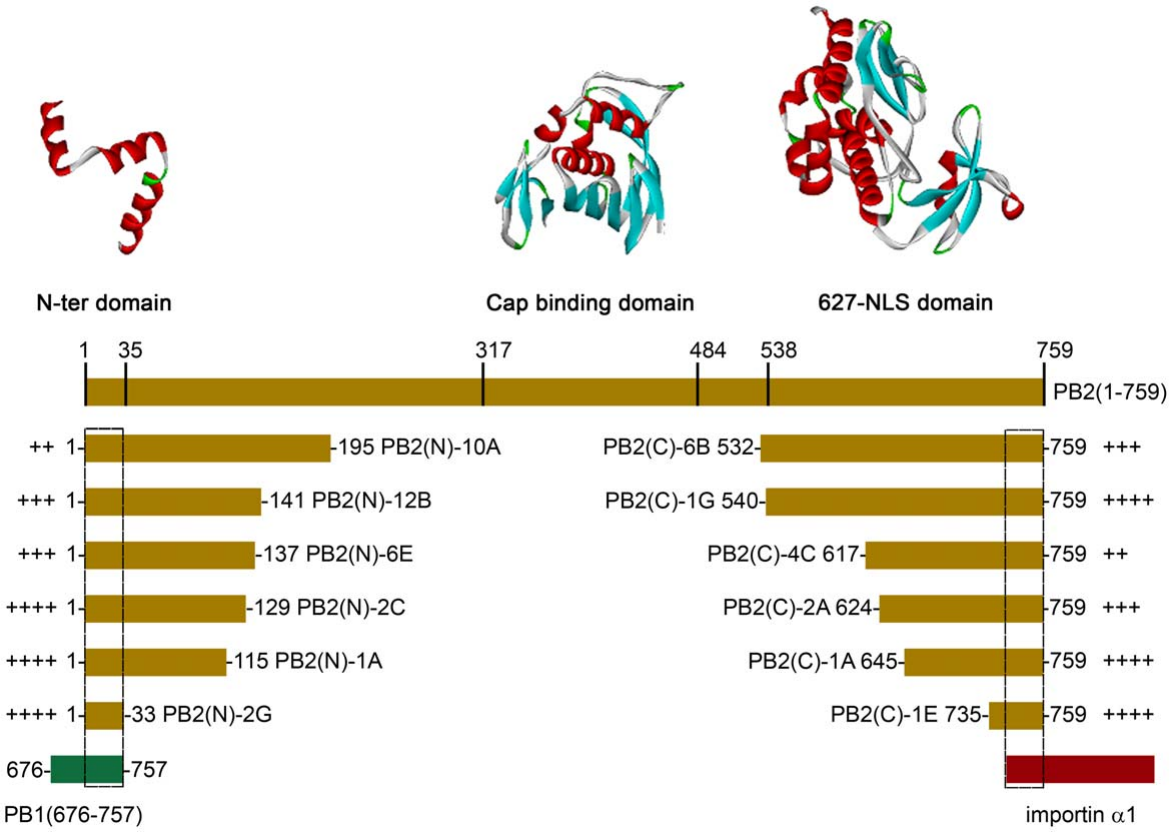
Alignment of PB2 constructs that co-purify with either PB1-(676–757) or importin α1. Constructs are aligned against the full-length PB2 polypeptide sequence with amino acid numbering shown. Indicated (+) are the yields of complex as assessed by Coomassie staining of SDS-PAGE gels shown in Fig. 4. Presented above are the known structural domains within PB2: the 627-NLS domain (PDB: 2vy6), cap-binding domain (PDB: 2vqz) and N-terminal PB1-binding domain (PDB: 2ztt; shown minus PB1 chain).

From the PB2 N-terminal truncation library co-expressed with importin α1, fifteen clones exhibiting complexes were retested in 30 ml cultures in 100 ml shake flasks with purification on identical Ni^2+^NTA agarose resin in a gravity column format (Fig. S3). Nine gave visible bands by SDS-PAGE with six identified as forming good quality stable and soluble protein complexes with importin α1 (Fig. 4A). Strong bands were obtained for both target and bait with similar intensities. The expression levels and purification protocols were not individually optimized at this stage so some *E. coli* contaminant proteins common to all purifications independent of bait or PB2 region were observed. The common band at 27 kDa, is likely the nickel-binding *E. coli* protein SlyD [47] since its binding was greatly reduced when using Co^2+^ affinity resin (data not shown), as previously reported [48]. Some minor proteolysis of the PB2 target was also observed: Constructs PB2(C)-1A (Fig. 4A) and PB2(N)-10A (Fig. 4B) appear partially degraded in the C-terminal region, the former having been described previously in the NLS region [39]. Yields were estimated at 4 mg per liter of culture which would be sufficient for structural studies. Inserts from the positive clones were sequenced to identify the truncation boundaries (Fig. 5). The soluble PB2 hits contained either the NLS domain (amino acids 678–759) [27] or larger 627-NLS double domain (amino acids 538–759) [39] that have both been shown previously to form complexes with separately purified importins upon mixing. One interesting exception was a very short construct (amino acids 735–759) that corresponds precisely the linear bipartite NLS peptide motif alone, the minimal set of amino acids for importin binding [27]. An early objective had been to determine whether co-expression of importin α1 would stabilise longer PB2 constructs containing the previously characterised interacting regions plus the crystallised upstream cap binding domain (amino acids 317–484; Fig. 5) [37]. No such clones were observed in agreement with the negative results obtained when these two regions were combined in a standard cloning strategy and expressed in the absence of importin α1 (data not shown).

**Figure 6:**
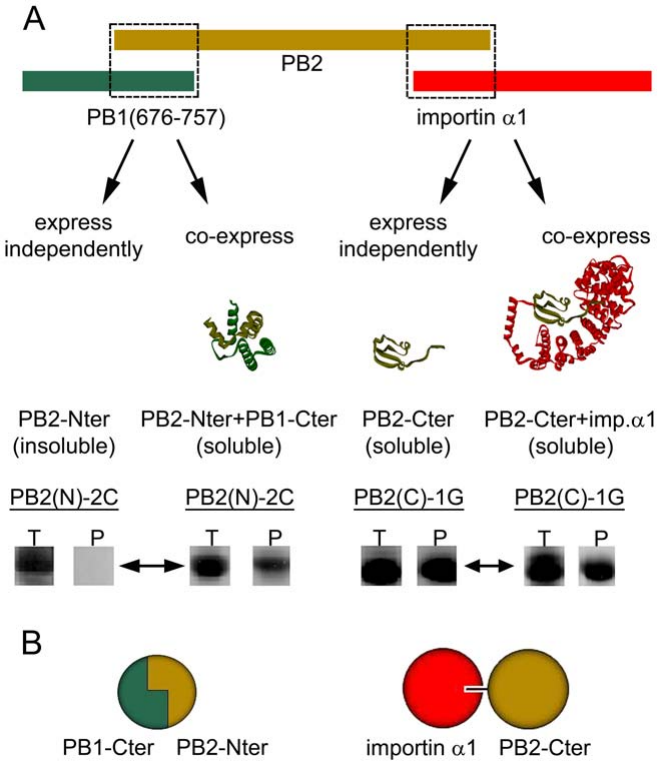
Formation of complexes of bait proteins and PB2 constructs. (A) PB2 N-terminal constructs are only soluble when co-expressed with a PB1 C-terminal fragment (amino acids 676–757), but insoluble when expressed independently (left); PB2 C-terminal constructs are soluble both when co-expressed with importin α1 or expressed independently (right). Annotations: T, total cell lysate; P, purified target protein. (B) Models for two types of protein complexes: PB1 C-terminal and PB2 N-terminal regions typify complexes generated from co-folded subunits (left); PB2 C-terminal regions and importin α1 associate as independently stable, pre-folded subunits (right).

From the PB2 C-terminal truncation library co-expressed with PB1-(676–757), 22 clones were selected for scale up (Fig. S3). Of these, 16 resulted in detectable purified complexes from which the six highest yielding are shown (Fig. 4B). By SDS-PAGE, bands of similar intensities were obtained indicating that the yield and complex quality would be potentially sufficient for structural studies. This PB1 fragment used as bait was identified previously using ESPRIT from a PB1 N-terminal truncation library and, although purifiable at low yields, was shown to be unstructured by NMR (data not shown). It is similar to a helical region characterised structurally as PB2 binding [43], [49]. Alignment of different PB1-binding fragments of PB2 (Fig. 5) revealed the minimal interacting region to be less than 40 amino acids, reflecting the ordered residues observed in the recently crystallised PB1–PB2 interaction region [43]. When these PB2 sub-constructs were expressed independently of the PB1 bait, five were totally insoluble as shown for clone 2C (Fig. 6A). The shortest peptide-sized PB2 construct (amino acids 1–33) was soluble; its short length may permit it to evade host proteases whilst it contains no hydrophobic core [43]. These data show that the formation of the PB2–PB1 complex stabilises PB2 when expressed in *E. coli* (Fig. 6A). In contrast, all the PB2 C-terminal constructs were soluble both in the presence and absence of importin α1 (Fig. 6A). Thus the complexes obtained by screening libraries of PB2 against these two different baits represent two common types of protein complexes (Fig. 6B): the PB1 C-terminal and PB2 N-terminal regions forms an inter-folded and stable structure while the PB2 C-terminal region and importin α1 dock together as pre-folded subunits.

## Discussion

Here we describe CoESPRIT, a library scale construct screening methodology that incorporates co-expression of bait proteins into our previously reported protocol for identifying soluble sub-constructs of a target protein [27], [28]. It allows identification of protein domains or fragments that interact with a fixed bait protein and is therefore similar in concept to deletion analyses by yeast-2-hybrid screening, but with the important distinction that the yields of purified protein are compatible with downstream studies such as crystallisation, NMR, biophysical methods (e.g. small angle X-ray scattering, analytical ultracentrifugation), as well as recombinant vaccine testing, that require multi-milligram quantities of material. Additionally, the *E. coli* host provides the option of labelling proteins with isotopes or heavy atoms as needed that is less straightforward with other organisms. Compatible plasmids were designed that permitted screening of the library of constructs in a strain prepared as competent cells co-expressing the bait. The first colony screening step identifies putatively soluble forms of the target that may or may not complex with the bait. As recently described [28], simultaneous analysis of fused N- and C-terminal peptide tags from colony signals of a truncated target provides a means to eliminate the out-of-frame constructs that are a majority species in DNA truncation libraries, together with in-frame gene products that are post-translationally degraded. A second step of purification screening of initial positives confirms the soluble phenotype of the target, and identifies library members that co-purify with the bait (Fig. 2). The main advantages of this approach are that expression of unsuspected or difficult-to-predict domains can be achieved without prior knowledge of the domain content of the target, and that folding-upon-binding effects can stabilise fragile or otherwise insoluble constructs where complex formation is required to form a hydrophobic core and protect the target from aggregation or proteolysis.

A standard and convenient screening capacity is approximately 27,648 clones that occupies seventy-two 384 well plates; this can be picked in two days and then arrayed onto a single membrane for expression screening. Such a capacity can be used to screen a library of a single target with high oversampling for unidirectionally truncated genes, or with about 5% coverage of total diversity for inserts truncated at both ends simultaneously [30]. Here we constructed two unidirectional truncation libraries (PB2 N-terminal and C-terminal truncation libraries) and screened them against 4 different baits, in parallel on the same membrane. At this level of sampling it would not be feasible to screen a target library against a bait library since the diversity of clones in such an experiment (tens of millions) would greatly exceed the screening capacity of the automation. However, a sparser sampling of both constructs randomised simultaneously might in some cases allow direct identification of complexes that could be further refined by subsequent steps of rescreening individually, or by limited proteolysis and mass spectrometry depending on the requirements of the downstream application. In a first step in this direction, we have demonstrated how a library-based screening strategy can permit identification of an optimal form of the bait protein amongst several similar candidates as shown by screening one PB2 library against three different PB1 fragments where only one of the latter proved competent for complex formation. Such coverage of construct diversity would be practically impossible using classic PCR cloning strategies due to issues of clone handling and cost of reagents. Here it is a relatively simple procedure because all constructs of a target are made in a single reaction tube and plasmid molecules clonally separated by bacterial transformation and robotic colony picking.

As a test case, we analysed truncation libraries of the influenza polymerase PB2 subunit where our earlier ESPRIT protocol had proved fruitful in identifying previously unknown domains for structural analyses [27], [37], [39]. Besides providing biologically interesting examples of co-folded and pre-folded subunits, an additional aim was to see whether previously uncharacterised constructs could be identified through expression of validated, potentially stabilising partners. The N terminus of PB2 has been shown recently by X-ray crystallography to co-fold with the C terminus of PB1 [43] whilst the C terminus contains a small domain that supports a NLS that has been shown, also by X-ray crystallography, to bind tightly to importin α nuclear import receptors (Fig. 5) [27], [39].

Investigations into the domain structures of the influenza polymerase have led to a series of new crystal structures by our lab and others, reviewed in [41], [42]. Here we have identified a series of constructs by empirical screening that form stable complexes with known partners validated recently through structural studies: PB2-importin α [27] and PB1–PB2 [43]. The availability of these structural data permits us to rationalise the results of the screen and the second step of re-expression of hits minus bait protein. Of the PB1–PB2 complexes identified, the smallest PB1-(amino acids 676–757)-PB2-(amino acids 1–33) is similar to the ordered residues in a recent crystal structure that shows the two polypeptides to intertwine, forming a structural module (Fig. 6) [43]. When expressing the subunits separately, the stability of the constructs is severely compromised. In contrast, the C-terminal PB2 constructs can be expressed in the absence of the importin α1 bait with no loss of solubility, consistent with their structural independence and previous studies showing that the proteins can be produced separately and complexes constituted by mixing (Fig. 6).

In summary, CoESPRIT is an efficient method for identification of purifiable soluble complexes at yields compatible with downstream studies. Interacting polypeptides are identified from small scale screening that, following scale up, should be validated by size exclusion chromatography or other biophysical techniques. They can be used directly in some applications (e.g. biophysical analyses or immunisation), or may require further refinement including limited proteolysis to remove unstructured regions, or tag removal prior to crystallisation trials. However these are standard procedures that are greatly facilitated by the availability of purified complexes as starting material. CoESPRIT, in common with other library methods including our original method employs the principles of directed evolution whereby a target gene encoding a poorly expressed target is incrementally truncated to generate random genetic diversity. Rare clones with improved solubility characteristics and able to form stable complexes with a co-expressed bait are isolated from the random library using a high-throughput, automated screening workflow based upon commonly accessible robotic systems. The level of coverage of construct diversity far exceeds that of standard high throughput cloning strategies and has particular advantages when the information on the target does not permit design of expression constructs. Although this method was designed to identify stable binary protein complexes, analysis of multiple subunit complexes could easily be achieved by combining the library vector with systems for simultaneous expression of several other subunits e.g. the multi-cassette ACEMBL system [50], the multi-plasmid pET-DUET system (Novagen) or polycistronic pST44 [51], [52]. Additionally, other types of partner such as chaperones for enhancing protein folding or modifying enzymes such as phosphatase and kinases can be expressed from the bait vector and will provide additional tools for challenging, difficult-to-express proteins.

## Materials and Methods

### Generation of plasmids for PB2 library construction

The PB2 gene from influenza virus strain A/Victoria/3/1975(H3N2) [53] was codon-optimised for expression in *E. coli* (Geneart, Regensburg, Germany) [27] and amplified using primers PB2-Synth-For3: 5′-GAGAT ATACA TATGG GCCAC CATCA TCACC ACCAT GATTA TGATA TTCCA ACTAC CGAGA-3′ and PB2-Rev: 5′- CCATT GTTCG ATGCA TTATT AATCG CCATA CGAAT ACGTT TGGTC GCGGT C-3′. The product was cloned into pYUM6002, a pET9a derivative from which non-essential DNA had been deleted, to give plasmid pYAN008 for N-terminal truncation (5′ DNA deletion) library construction (Fig. S1A). Similarly, the PB2 gene was amplified using PB2-For: 5′-CTAGG ACGTC GATGG AACGC ATTAA AGAAC TGCGC AACCT G-3′ and PB2-Synth-Rev3: 5′- CCCGT TCATT ATTCG TGCCA TTCGA TTTTC TGAGC CTCGA AGATG TCGTT CAGCC CACCG-3′ and cloned into alternative restriction sites of pYUM6002 to give plasmid pYAN009 for C-terminal truncation (3′ DNA deletion) library construction (Fig. S1A). *E. coli* Mach1 T1 cells (Invitrogen, Carlsbad, CA, USA) were used for efficient recovery of ligation products. Enzymes were purchased from New England Biolabs (Beverly, MA, USA) with primers synthesised by Invitrogen.

### Generation of plasmids for bait protein expression

The gene encoding *E. coli* codon optimised importin α1 Hu Imp-α1 [KPNA2; Uniprot: P52292, residues 60–529] (Geneart, Regensburg, Germany) was amplified using two overlapping forward primers FLAG-TEV-For1: 5′-GGAAT TCCAT ATGGG CGACT ACAAG GACGA CGATG ACAAG GATTA TGATA TTCC-3′, and FLAG-For2: 5′-CAAGG ACGAC GATGA CAAGG ATTAT GATAT TCCAA CTACC GAGAA TTTGT ATTTT CAGG-3′ with reverse primer importin-Rev1: 5′-ATAAG AATGC GGCCG CTCAT TAAAA ATTAA AGGTG CCCGG CGCAC CATCC-3′ to add an N-terminal FLAG tag peptide (DYKDDDDK) before the bait protein. The importin α1 gene was cloned into *Nde*I/*Not*I sites of the vector pLIC-SGC1 that is a pACYC184 derivative expressing rare *E. coli* tRNA genes into which a T7 expression cassette had been inserted (Structural Genomics Consortium, Toronto, Canada), forming plasmid pYAN010 (Fig. S1B). *E. coli* BL21 (DE3) cells were transformed with pYAN010, cultured in LB with chloramphenicol 30 µg/ml and used to prepare electrocompetent cells. The DNA fragments encoding PB1 C-terminal sub-constructs corresponding to amino acid sequences 586–696, 676–757 and 576–757 (Uniprot: 31341) were amplified by PCR, and cloned in the same way to generate plasmids pYAN011, pYAN012 and pYAN013 containing FLAG tagged PB1 bait proteins.

### Construction of PB2 random truncation libraries

A protocol using exonuclease III and mung bean nuclease was employed to construct incremental truncation libraries using a modification of a previously described protocol [27]. High quality plasmid was first prepared from 200 ml overnight culture of cells by standard alkaline lysis and phenol-chloroform extraction protocols, then further purified using a miniprep Kit (Qiagen, Valencia, USA). Ten micrograms of plasmid pYAN008 were digested to completion with *Aat*II (yielding an exonuclease III insensitive end adjacent to the promoter) and *Asc*I (yielding an exonuclease sensitive end adjacent to the gene insert), both sites being located upstream of the target gene. Four micrograms of purified, linearised plasmid were incubated in reaction buffer (1× Buffer 1 New England Biolabs supplemented with 30 mM NaCl) and 400 U of exonuclease III, in a final volume of 120 µl at 22°C. To ensure even fragment distribution, 0.5 µl of the reaction was removed every 30 s over 2 h and pooled in a tube containing 200 µl of 3 M NaCl on ice. The quenched reaction was denatured at 70°C for 20 min and DNA was purified using Nucleospin Extract II kit (Macherey-Nagel, Düren, Germany). In order to remove the 5′ overhang after exonuclease III digestion, the DNA was incubated with 5 U of mung bean nuclease in a final volume of 55 µl at 30°C for 30 min, then purified with the Nucleospin Extract II kit. The ends of the DNA molecules were polished by incubation with *Pfu* polymerase (Stratagene) in a final volume of 50 µl (1×*Pfu* polymerase native buffer, 2.5 mM dNTPs and 5 U of enzyme) at 72°C for 20 min. Then the reaction was electrophoresed in a 0.5% agarose gel and slices of gel containing linearised plasmid with inserts of size 0–700, 700–1400 and 1400–2100 bp were excised. DNA fragments were recovered from the gel using QIAEXII resin (Qiagen) and re-circularised using T4 DNA Ligation Kit (Roche, Mannheim, Germany); these were then used to transform *E. coli* Mach1 T1 competent cells (Invitrogen). Transformation mixes were recovered in SOC medium and plated on LB agar (supplemented with kanamycin 50 µg/ml) in 22 cm QTrays (Genetix, UK). After overnight growth at 37°C, approximately 4.0×10^4^ colonies for each sub-library were scraped from the agar respectively, then resuspended in phosphate buffered saline (PBS) buffer. The plasmid DNA mixture was extracted from the cell pellets using a miniprep kit.

A comprehensive random PB2 C-terminal truncation library was constructed using an almost identical approach, but differing in cleavage of pYAN009 with *Nsi*I and *Not*I located at the 3′ end of the PB2 gene insert that had been cloned in frame with a 5′ sequence encoding N-terminal TEV-cleavable hexahistidine tag. Approximately 5×10^4^ colonies for each sub-library were plated and pooled for DNA extraction.

### Preparation of colony arrays for expression screening

Competent cells were prepared of *E. coli* BL21 (DE3) harbouring pYAN010 FLAG-bait expression vectors and transformed with target sub-libraries. The libraries were plated on 22 cm LB agar QTrays (supplemented with kanamycin and chloramphenicol) at a density of approximately 3,000 colonies per plate, and incubated overnight at 30°C. Then totally 9,216 colonies from both PB2 N-terminal and C-terminal truncation libraries were isolated using a colony-picking robot (KBiosystems, Basildon, UK) into 384 well plates containing 70 µl TB medium per well (supplemented with kanamycin and chloramphenicol). Liquid cultures were grown overnight at 37°C in a HiGro shaker incubator (Genomic Solutions, Harvard, USA), and then all clones of each library were arrayed robotically onto a nitrocellulose membrane (Amersham, Arlington Heights, USA) on LB agar plates supplemented with antibiotics. Plates were incubated overnight at 25°C until colonies on the membrane were just visible. Then the membrane was carefully moved onto a fresh LB agar plate (supplemented with antibiotics, 0.1 mM IPTG and 50 µM biotin) to induce recombinant protein expression within the colonies at 30°C for 4 h. In the same way, plasmid DNAs extracted from PB2 C-terminal truncation sub-libraries were transformed into *E. coli* BL21 (DE3) stains harbouring pYAN011, pYAN012 and pYAN013 and processed as above.

### Identification of putative soluble protein-expressing clones

Membranes were incubated for 4 h at 30°C, lifted from the inducing agar and laid over filter paper soaked in denaturing buffer (0.5 M NaOH, 1.5 M NaCl) for 10 min at room temperature. The membranes were neutralised 2×5 min in neutralisation buffer (1 M Tris-HCl, pH 7.5, 1.5 M NaCl) and then for 15 min in 2×SSC buffer [44]. The cellular debris of colonies on the membrane were carefully removed with a glass spreader, and the membrane was blocked overnight in Superblock (Pierce, Chicago, USA) at 4°C. The membranes were washed 3 times with PBS-T (PBS with 0.05% Tween 20) buffer for 5 min each, and incubated in 50 ml of PBS-T containing 16 µl anti-hexahistidine antibody (Amersham) for 1 h at 4°C. After washing with PBS-T buffer, the membranes were further incubated in 50 ml of PBS-T containing 10 µl streptavidin Alexa Fluor 488 (Invitrogen) and 50 µl Alexa Fluor 532 goat anti-mouse IgG (Invitrogen) for 1 h. After washing with PBS-T buffer, the membranes were scanned with a Typhoon 9400 fluorescence imager (Amersham) for hexahistidine tag and biotin acceptor peptide signal intensities respectively. Signals from the array were quantified from digitised images using Visual Grid software (GPC Biotech, Waltham, USA) and data exported to Microsoft Excel for analysis. Clones were sorted according to hexahistidine signals and those exhibiting no clear signal were eliminated from further analyses. Those that remained were ranked according to their biotinylation signals in order to identify putatively soluble clones.

### High-throughput screening of co-expressed and co-purified protein complexes

The most intensely biotinylated 96 clones from each library were selected for small-scale protein expression at 4 ml scale in 24 well plates with TB medium supplemented with kanamycin and chloramphenicol. After the cultures reached OD_600_ = 0.8, 0.1 mM IPTG and 50 µM biotin were added to induce protein expression and enhance protein biotinylation respectively. After overnight induction at 25°C, the cells were pelleted by centrifugation, and resuspended in 4 ml of sphaeroplast buffer (20 mM Tris pH 8.0, 140 mM NaCl, 20% sucrose and 1 mg/ml lysozyme). The sphaeroplasted cells were harvested by centrifugation and then resuspended in 800 µl lysis solution per well containing 10 mM Tris pH 7.5, 0.5% Brij, 0.25 U/µl Benzonase (Novagen, San Diego, USA) and 0.8 µl Protease Inhibitor Cocktail (Sigma, Saint Louis, USA). Ninety-six protein samples were simultaneously purified on a liquid handling robot [33] with samples loaded into a 96 well filter plate supplemented with 60 µl Ni^2+^NTA agarose (Qiagen) in each well and mixed by rotation at 4°C for 30 min. The samples were then washed with washing buffer (50 mM phosphate buffer, pH 7, 140 mM NaCl, 5 mM imidazole) and eluted with elution buffer (50 mM phosphate buffer, pH 7, 140 mM NaCl, 300 mM imidazole). The 96 samples were subjected to SDS–PAGE, and then electroblotted onto a nitrocellulose membrane. The membrane was first probed with a 1∶1000 dilution of a rabbit anti-FLAG antibody (Sigma) and then after a wash step, incubated with a mix of a 1∶1000 dilution of anti-rabbit IgG 633 (Invitrogen) and 1∶1000 dilution of Streptavidin 488. After washing with PBS-T, the membrane was scanned using a fluorescence imager to analyse FLAG and biotin acceptor peptide signals.

### Scale-up expression and purification of selected clones

Plasmids were purified from the clones identified as soluble co-expression hits, and the DNA inserts characterised by sequencing to identify domain boundaries. The positive clones were grown at 37°C in 30 ml of TB media supplemented with kanamycin and chloramphenicol. At OD_600_ = 0.8, 0.1 mM IPTG and 50 µM biotin were added. After induction at 20°C overnight at 100 rpm, the cells were harvested by centrifugation and 1 g of each cell pellet was resuspended in 2 ml of resuspension solution containing 50 mM phosphate buffer, pH 7.0, 0.25 U/µl Benzonase and 2 µl Protease Inhibitor Cocktail (Roche) and lysed by sonication. After centrifugation, the supernatant was collected and loaded into columns supplemented with 100 µl Ni^2+^NTA resin, washed (50 mM phosphate buffer, pH 7.0, 140 mM NaCl, 50 mM imidazole), and eluted (50 mM phosphate buffer, pH 7.0, 140 mM NaCl, 300 mM imidazole) to obtain proteins that were analysed by SDS–PAGE.

## Supporting Information

Figure S1 **Schematic representation of plasmids used for truncation library construction and soluble complex screening.** (A) Plasmids designed for library construction (pYAN008 and pYAN009). The plasmid pYAN008 has restriction sites (*Aat*II and *Asc*I) designed for creating incremental truncation libraries from the 5′ end of the target gene; a hexahistidine tag site and a TEV protease cleavage site precede PB2 out-of-frame, and a biotin acceptor peptide sequence is fused in-frame downstream. For plasmid pYAN009 used for creating incremental truncation libraries from the 3′ end of the target gene, *Not*I and *Nsi*I sites are positioned downstream of the gene insert which is fused in-frame with a hexahistidine tag and TEV protease cleavage site. Downstream of the insert is a biotin acceptor peptide that is out of frame with the gene. For both plasmids, a short sequence of DNA separates restriction enzymes and a different reading frame prevents read through from the tag sequence into the gene (…//…). (B) The plasmid used for bait protein expression as fusions with a TEV protease cleavable N-terminal FLAG tag. The *ileX*, *argU*, and *leuW genes* in the plasmid encode rare *E. coli* tRNAs.
(DOCX)

Figure S2 **Frequency histogram analysis of total biotinylation signals from the subunit expression libraries.** (A) PB2 N-terminal truncation library co-expressed with importin α1, and (B) PB2 C-terminal truncation library co-expressed with PB1 (amino acids 676-757). The chart displays the frequency of clones according to their streptavidin signals.The Y-axis is the frequency and X-axis the binned signal intensities. The 96 most intensely biotinylated clones selected for subsequent expression tests exhibited higher signals than the background level from the endogenous BCCP protein.
(DOCX)

Figure S3 **Summary of processing of each library through purification screening and analysis steps of CoESPRIT.** The numbers of clones passing through each step are indicated.
(DOCX)
