# Supplementary figures and images for: CoESPRIT: A Library-Based Construct Screening Method for Identification and Expression of Soluble Protein Complexes
Source: PLoS One. 2011 Feb 22;6(2):e16261. doi: 10.1371/journal.pone.0016261 (PMC3043051; doi:10.1371/journal.pone.0016261)

**Figure S1**

**
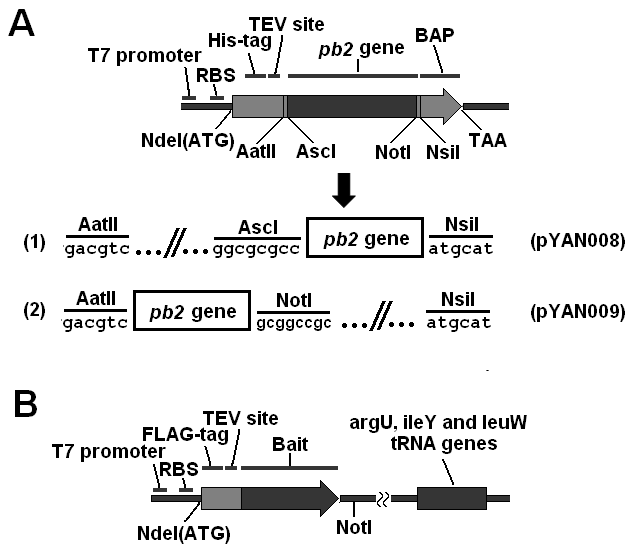
**

Supplement: Figure S1 — Schematic representation of plasmids used for truncation library construction and soluble complex screening. (A) Plasmids designed for library construction (pYAN008 and pYAN009). The plasmid pYAN008 has restriction sites (AatII and AscI) designed for creating incremental truncation libraries from the 5′ end of the target gene; a hexahistidine tag site and a TEV protease cleavage site precede PB2 out-of-frame, and a biotin acceptor peptide sequence is fused in-frame downstream. For plasmid pYAN009 used for creating incremental truncation libraries from the 3′ end of the target gene, NotI and NsiI sites are positioned downstream of the gene insert which is fused in-frame with a hexahistidine tag and TEV protease cleavage site. Downstream of the insert is a biotin acceptor peptide that is out of frame with the gene. For both plasmids, a short sequence of DNA separates restriction enzymes and a different reading frame prevents read through from the tag sequence into the gene (…//…). (B) The plasmid used for bait protein expression as fusions with a TEV protease cleavable N-terminal FLAG tag. The ileX, argU, and leuW genes in the plasmid encode rare E. coli tRNAs. (DOCX) [file pone.0016261.s001.docx]

**Figure S2**

**
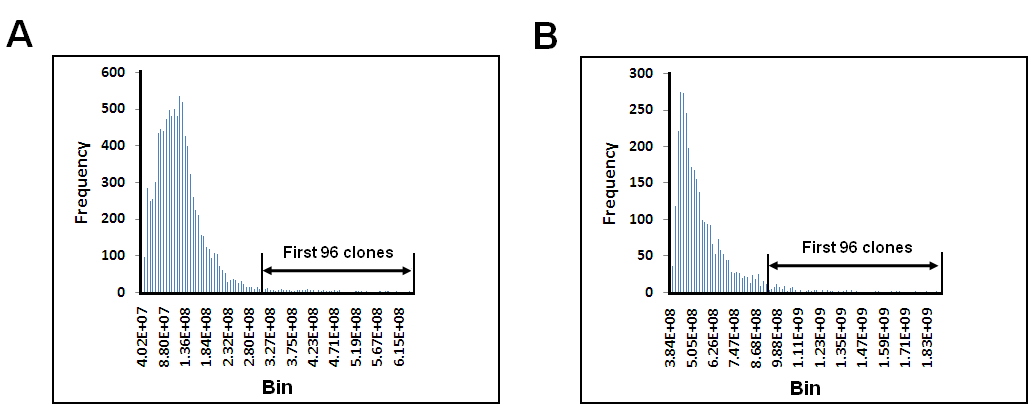
**

Supplement: Figure S2 — Frequency histogram analysis of total biotinylation signals from the subunit expression libraries. (A) PB2 N-terminal truncation library co-expressed with importin α1, and (B) PB2 C-terminal truncation library co-expressed with PB1 (amino acids 676-757). The chart displays the frequency of clones according to their streptavidin signals.The Y-axis is the frequency and X-axis the binned signal intensities. The 96 most intensely biotinylated clones selected for subsequent expression tests exhibited higher signals than the background level from the endogenous BCCP protein. (DOCX) [file pone.0016261.s002.docx]

**Figure S3**

**
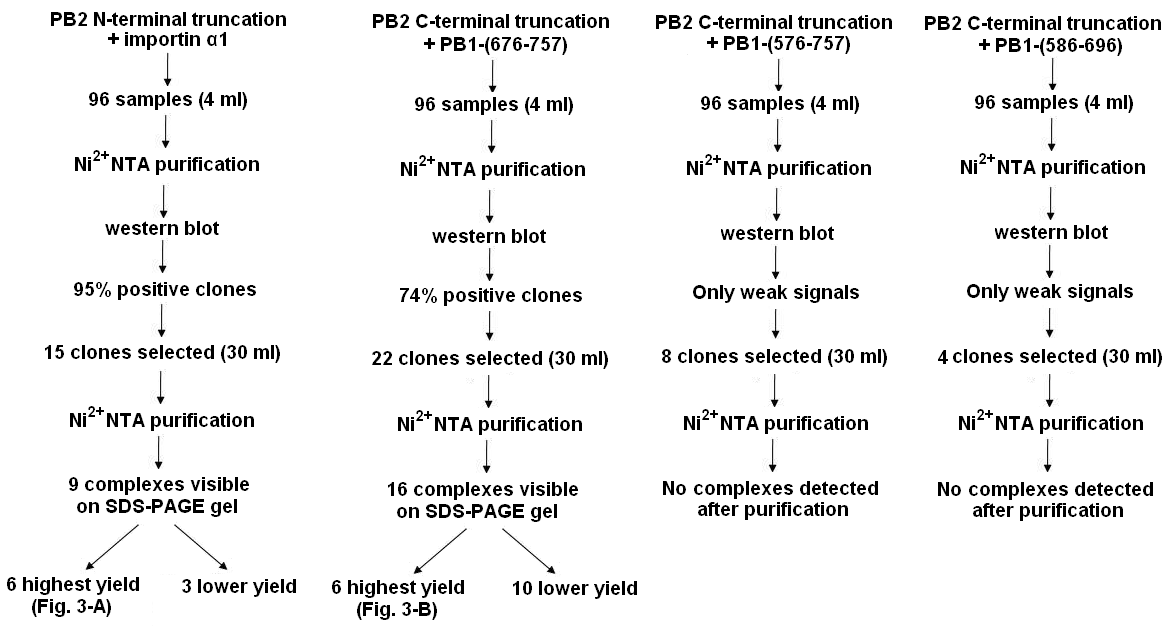
**

Supplement: Figure S3 — Summary of processing of each library through purification screening and analysis steps of CoESPRIT. The numbers of clones passing through each step are indicated. (DOCX) [file pone.0016261.s003.docx]
